# Plasma iron concentrations and systemic inflammatory response syndrome in neonatal foals

**DOI:** 10.1111/jvim.15770

**Published:** 2020-04-16

**Authors:** Júlia Sanmartí, Lara Armengou, Judit Viu, Eduardo Alguacil, Sandra Civit, José Ríos, Eduard Jose‐Cunilleras

**Affiliations:** ^1^ Servei de Medicina Interna Equina, Departament de Medicina i Cirurgia Animals Facultat de Veterinària, Universitat Autònoma de Barcelona Barcelona Spain; ^2^ Unitat Equina, Fundació Hospital Clínic Veterinari Universitat Autònoma de Barcelona Barcelona Spain; ^3^ Hospital Veterinario Sierra de Madrid, 28750‐San Agustín de Guadalix Madrid Spain; ^4^ Uplands Way Vets Low Road, Bressingham, IP22 2AA Diss Norfolk UK; ^5^ Ganaderia JM Barcelona Spain; ^6^ Unitat de Bioestadística, Facultat de Medicina, Universitat Autònoma de Barcelona Barcelona Spain

**Keywords:** critical care, monitoring, neonatal foals, plasma iron, systemic inflammatory response syndrome

## Abstract

**Background:**

Sparse information regarding plasma iron concentration in neonatal foals and its utility as an inflammatory marker in this population has been published.

**Objectives:**

To determine the physiologic plasma iron concentration in neonatal foals. To assess its utility as an inflammatory marker to predict systemic inflammatory response syndrome (SIRS) and as a prognostic marker.

**Animals:**

Forty‐seven ill neonatal foals admitted to a referral equine hospital were divided in 2 groups based on the SIRS criteria (24 SIRS and 23 non‐SIRS). Two control groups of 43 hospital and 135 stud farm healthy neonatal foals were also included.

**Methods:**

Observational prospective study. Data were summarized by mean and its 95% confidence interval and absolute frequency and percentage for quantitative andqualitative variables. One‐way ANOVA, ANCOVA (group and age effects) and Dunnett as posthoc analysis were used to compare plasma iron concentration among groups.

**Results:**

Neonatal foals with SIRS did not have had any statistically significant different plasma iron concentrations compared to non‐SIRS (*P* = .56) and stud farm control group (*P* = .99), 172.8 μg/dL (95% CI; 126.0‐219.6), 193.1 μg/dL (139.1‐247.2), and 181.8 μg/dL (171.3‐192.4), respectively. Plasma iron concentration had a large variability in healthy neonatal foals, and was negatively correlated with age in hospital controls (rho = −0.387) and sick neonatal foals (rho = −0.598) (*P* < .001).

**Conclusions and Clinical Importance:**

Plasma iron was not a useful marker of SIRS in neonatal foals and was not associated with outcome.

**Abbreviations**
RBC: red blood cell
SIRS: systemic inflammatory response syndrome

## 1 INTRODUCTION

Sepsis in neonatal foals is associated with higher death rate compared with other medical conditions affecting neonatal foals.^1^ Early detection of systemic inflammation is essential in order to provide appropriate treatment.^2^ The original systemic inflammatory response syndrome (SIRS) criteria in neonatal foals have a significant association with prediction of sepsis and nonsurvival.^3^

The SIRS term provides a reference for the complex findings that resulted from a systemic activation of the innate immune response, regardless of the cause.^4^ Systemic inflammatory response syndrome describes the clinicopathologic effects of the inflammatory response to a variety of insults, including bacterial infection, endotoxemia, ischemia, hypoxia, trauma, and burns.^5^ The original SIRS criteria used to describe this clinical syndrome in neonatal foals included presence of 2 or more of: (1) hyper‐ or hypo‐thermia (rectal temperature >39.2°C or <37.2°C); (2) leukocytosis or leukopenia (peripheral white blood cell count >12.5 × 10^3^/μL or <4 × 10^3^/μL) or > 10% immature (“band”) neutrophils; (3) tachycardia (>120 beats/min); and (4) tachypnoea (> 30 breaths/min).^3^, ^5^, ^6^, ^7^, ^8^ The SIRS criteria is much simpler and faster to use than sepsis scores and might serve as a more rapid screening tool for sepsis in neonatal foals. A positive result to the original SIRS criteria has a sensitivity of 60% and specificity of 69% to predict sepsis when compared to other proposed SIRS criteria^3^, ^9^, ^10^ and could be more useful for predicting nonsurvival associated with sepsis.^3^ Based on the human pediatric literature, an updated SIRS score for foals was proposed but it does not provide an improved ability in predicting sepsis.^3^, ^11^ SIRS criteria for foals of the aforementioned study required the presence of at least 3 of: (1) fever or hypothermia, (2) tachycardia, (3) tachypnea, (4) leukocytosis, leukopenia, >5% band neutrophils, (5) venous blood lactate concentration, or (6) venous blood glucose concentration; at least one of which had to be abnormal temperature or leukocyte count.^9^

In adult horses, plasma iron concentration acutely decreases in cases with systemic inflammation.^2^ Low iron and high fibrinogen plasma concentrations are both sensitive indicators of systemic inflammation in horses, with sensitivity of 90 and 82%, respectively.^12^ Rapid development of hypoferremia is particularly valuable during the earliest phases of infection, before other components of innate and adaptive immunity are mobilized.^2^

Red blood cell (RBC) mass in human neonates is highly variable, because of changes in both the mass and the composition of RBCs occurring during the transition from the intra uterine to the extra uterine environment.^13^ Sparse information is published about iron in neonatal foals.^14^, ^15^

The objectives of this study were to determine the physiologic values of plasma iron concentrations in neonatal foals (<14 days old), and to assess its utility as both an early inflammatory marker to predict SIRS and a prognostic marker in sick neonatal foals.

## 2 MATERIALS AND METHODS

### 2.1 Animals

Neonatal foals (<14 days old) referred to the Equine Unit of the Fundació Hospital Clínic Veterinari of the Universitat Autònoma de Barcelona from January 2005 to December 2011 were included if a blood sample was taken before any treatment was administered. Forty‐seven ill neonatal foals were divided in 2 groups: SIRS (n = 24) and non‐SIRS (n = 23). The SIRS group was defined as meeting 2 or more of: (1) hyper‐ or hypo‐thermia (rectal temperature >39.2°C or <37.2°C), (2) leukocytosis or leukopenia (peripheral white blood cell count >12.5 × 10^3^/μL or <4 × 10^3^/μL) or >10% immature (“band”) neutrophils, (3) tachycardia (>120 beats/min), and (4) tachypnea (>30 breaths/min).^3^, ^5^, ^6^, ^7^, ^8^

Foals that did not meet the SIRS criteria were classified as non‐SIRS. The hospital control group (n = 43) included healthy neonatal foals admitted to the same referral equine hospital from 2005 to 2011 that had a normal physical examination and were accompanying their sick dams. Another control group was added, including 135 healthy neonatal foals from a nearby endurance stud farm over the 2017 foaling season. These were 24‐48 hour‐old foals with history of normal foaling, normal physical exam at birth and serum immunoglobulin G (IgG) concentration above 800 mg/dL within 24‐48 hours postpartum.

Sick foals (ie, SIRS and no SIRS groups) were also classified according to outcome into survivors (n = 29) and nonsurvivors (n = 18). The nonsurvivor group included both cases of natural deaths (n = 4) and euthanized because of poor prognosis (n = 14). None of the foals included in the nonsurvivor group was euthanized because of financial restraints.

### 2.2 Blood sampling and measured variables

Blood samples from ill neonatal foals were collected at admission in ethylenediaminetetraacetic acid (EDTA) and lithium heparin tubes. Similarly, blood samples from healthy hospital neonatal foals (hospital control group) were collected at different times during hospitalization, usually within the first 48 hours of admission. Finally, blood samples from stud farm control group were collected from 24 to 48 hours after birth.

Blood collected in EDTA from sick foals was used to determine manual hematocrit, total protein by refractometry, manual fibrinogen by heat precipitation method,^16^ and also to perform Diff‐Quick stained blood smears and complete cell blood count using automated hematology analyzers (Advia 120, Siemens Health Care Diagnostics SL, Barcelona, Spain; LaserCyte, Idexx laboratories, Inc, Netherlands). In the hospital control group, in addition to a normal physical examination, hematocrit, total protein and fibrinogen concentrations were determined to support that foals were healthy to be included in this group (data not shown). In the stud farm control group, serum IgG and plasma iron concentration were the only measured variables. Serum IgG was determined using a portable quantitative analyzer (DVM Rapid TestTM II ‐ Multi‐Test Analyzer Tests, Florida) for the stud farm control group and semiquantitative turbidimetric test (ZnSO_4_ test) for the hospital control group and sick group. Plasma iron concentration was measured from lithium heparin plasma samples with an automated chemistry analyzer (Olympus AU400, Hamburg, Germany) using a conventional enzymatic colorimetric assay (Olympus System Reagent, Beckman Coulter, Galway, Ireland) by the TPTZ [2,4,6‐Tri‐(2‐piridil)‐5‐triacina] as chromogen.

### 2.3 Data analysis

The main objective of this study was to establish the usefulness of iron concentration for diagnosis and prognosis in neonatal foals and obtain an estimate of limits of abnormality (ie, reference range) in neonatal foals. These limits were estimated by calculation of individual 95% confidence intervals (95% CI) (ie, from the SD as a measure of variability), and absolute range (minimum and maximum) for plasma iron concentration for each group, defined as sick, hospital control and stud farm control groups. One‐way ANOVA and Dunnett as posthoc analysis of group comparisons detected differences between groups. To evaluate the effect of age on plasma iron concentration, ANCOVA analysis of group as independent factor and age as covariate was performed. Additionally, using *t* test for independent groups, differences between SIRS, non‐SIRS, and outcome were explored from 95% CI of mean (ie, using SE as measure of variability). Cutoffs of less than 59, 79, and 105 μg/dL were tested in order to evaluate the utility of external limits of plasma iron concentration and a Fisher's exact test was performed. Other variables were described by mean and their 95% CI and absolute frequency and percentage for quantitative and qualitative variables, respectively. All statistical analyses were performed using a statistical software package (SPSS version 25, SPSS Inc, Chicago, Illinois) and all analyses were performed with a 2‐sided Type I error of 5%.

## 3 RESULTS

A total of 225 neonatal foals (<14 days old) were included in the study. Forty‐seven out of 225 were sick neonatal foals with mean age of 3.0 days (95% CI; 2.2‐3.9 days). Sick neonatal foals were divided into SIRS group (n = 24), mean age 3.5 days (95% CI; 2.2‐4.8 days), and non‐SIRS group (n = 23), mean age 2.6 days (95% CI; 1.5‐3.7 days). Forty‐three were healthy foals admitted to the hospital, with mean age 4.5 days (95% CI; 3.4‐5.6 days) and the 135 healthy neonatal foals left were from a stud farm with mean age 1.5 days (95% CI; 1.5‐1.5 days).

**FIGURE 1:**
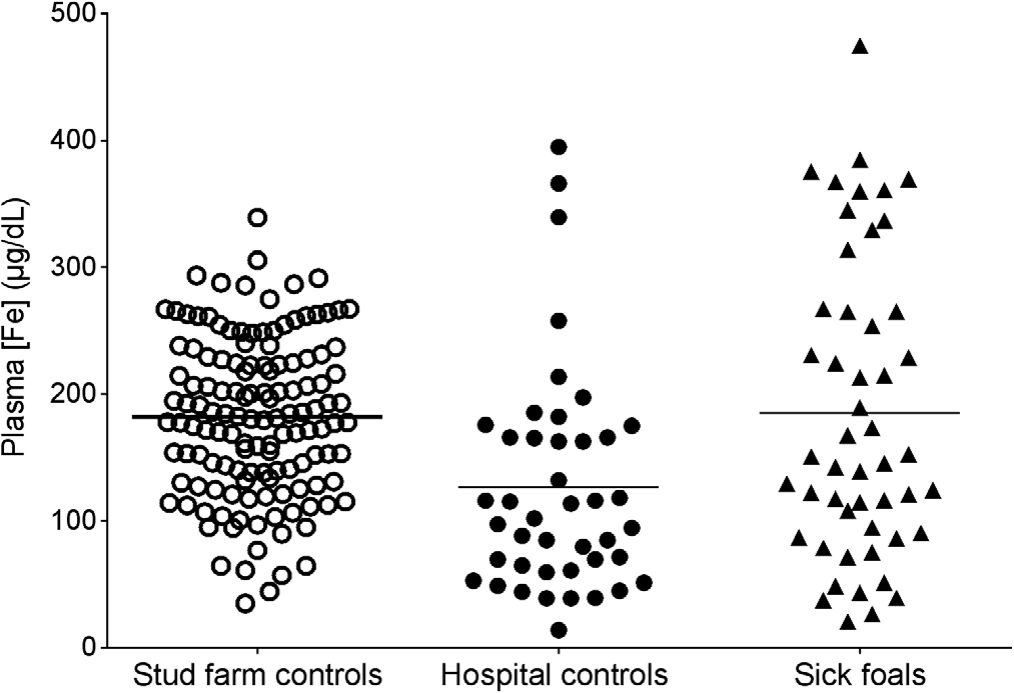
Scatterplot of plasma iron concentrations (μg/dL) in stud farm controls (open circles), hospital controls (solid circles), and sick neonatal foals (solid triangles). Horizontal line indicates mean plasma iron concentration for each group

**FIGURE 2:**
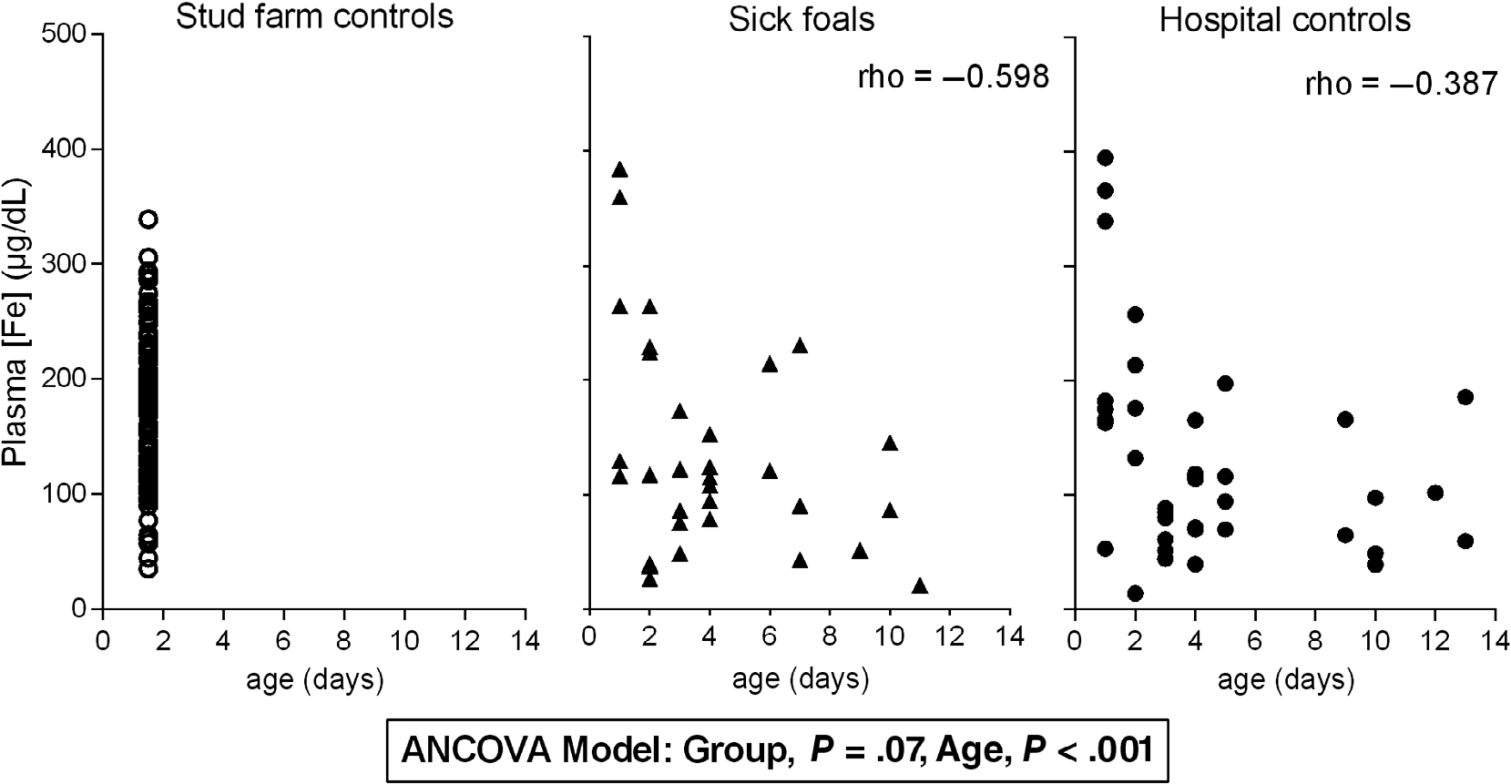
Scatterplot of the relationship of plasma iron concentrations (μg/dL) and age (days) in healthy [stud farm controls (open circles) and hospital controls (solid circles)] and sick neonatal foals (solid triangles)

The 95% CI limits of plasma iron concentration of the sick group were similar to the stud control group (*P* = .99) (Table 1; Figure 1). The hospital control group had significantly lower 95% CI limits of plasma iron concentrations and lower mean plasma iron concentration than sick group (*P* = .002) (Table 1; Figure 1). With ANCOVA analysis, there was no significant difference between the hospital control and sick foals (*P* = .06) but age effect was present (*P* = .001; Figure 2). A negative moderate correlation with age was seen in the hospital control group (rho = −0.387) and in the sick group (rho = −0.598), so the higher the age the lower the iron concentration. Greater variability and higher iron plasma concentrations were specially seen in the first 3 days of life (Figure 2).

**TABLE 1:** Individual 95% confidence intervals

		Stud farm controls	Hospital controls	Sick foals
Iron (μg/dL)	Ind (95% CI)	(58.8; 305)	(0.0; 303)	(0.0; 420)
	Range	35.0‐339.1	13.9‐394.8	20.7‐474.9
	Mean (95% CI)	181.8^a^ (171.3; 192.4)	126.1^b^ (99.2; 153.1)	183.2^a^ (148.5; 217.8)
	N	135	43	47
Age (days)	Mean (95% CI)	1.5^c^ (1.5; 1.5)	4.5^c^ (3.4; 5.6)	3.0^c^ (2.2; 3.9)
	Range	1.5‐1.5	1‐13	1‐11
	N	135	43	47

*Note:* Data presented as individual 95% confidence interval (Ind (95% CI)); range, (minimum and maximum); mean (95% CI), 95% confidence intervals of the mean; N, number of foals.

a Indicates no significant difference between groups (*P* > .5).

b Indicates significant difference between groups (*P* < .05).

c Indicates significant difference between groups with the ANCOVA model (*P* < .001).

**FIGURE 3:**
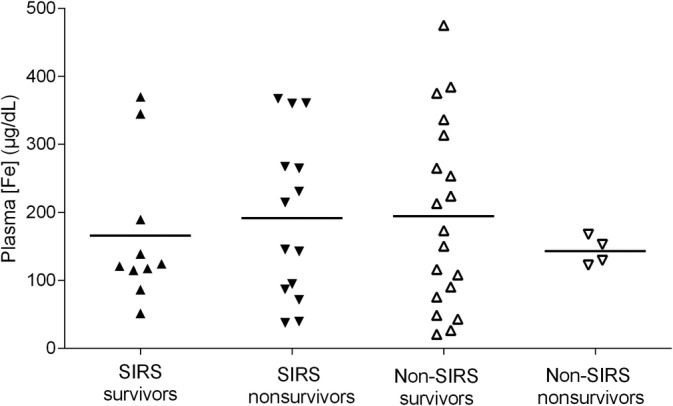
Scatterplot of plasma iron concentrations (μg/dL) in sick neonatal foals: SIRS survivors (upright solid triangles), SIRS nonsurvivors (upside down solid triangles), non‐SIRS survivors (upright open triangles) and non‐SIRS nonsurvivors (upside down open triangles). Horizontal line indicates mean plasma iron concentration for each group

Both individual 95% CI and absolute range of plasma iron concentration of SIRS group overlapped with the non‐SIRS group (Table 2; Figure 3). Plasma iron concentration of non‐SIRS group (193.10 μg/dL) was not statistically significant different to SIRS group (172.77 μg/dL) (*P* = .56; Figure 3).

**TABLE 2:** Plasma iron concentration in sick neonatal foals with SIRS versus non‐SIRS

		Non‐SIRS	SIRS	*P*‐value
	N	24	23	
Iron (μg/dL)	Ind (95% CI)	193 (0.0; 458)	173 (0.0; 397)	
	Range	20.7–474.9	37.5‐369.6	
	Mean (95% CI)	193.1 (139.1; 247.2)	172.8 (126.0; 219.6)	.56
Iron < 59 μg/dL	No	**20 (83.3%)**	20 (87.0%)	1.0
	Yes	4 (16.7%)	**3 (13.0%)**	
Iron < 79 μg/dL	No	**19 (79.2%)**	19 (82.6%)	1.0
	Yes	5 (20.8%)	**4 (17.4%)**	
Iron < 105 μg/dL	No	**18 (75.0%)**	16 (69.6%)	.75
	Yes	6 (25.0%)	**7 (30.4%)**	

*Note:* Data presented as individual 95% confidence interval (Ind 95% CI); range, (minimum and maximum); Mean (95% CI), 95% confidence intervals of the mean; N, number of foals.

Sick neonatal foals were divided according to outcome into survival group (n = 29) and nonsurvival group (n = 18) (Figure 3). Survival group showed mean plasma iron concentration of 184.58 μg/dL with 95% CI limits of concentration (136.71‐232.46 μg/dL) and nonsurvival group 180.84 μg/dL with 95% CI limits of concentration (127.48‐234.20). Plasma iron concentration was not statistically associated with outcome (*P* = .92).

External cutoff values (less than 59, 79, and 105 μg/dL) were applied for plasma iron concentration with no statistically significant differences between those (Table 2).

## 4 DISCUSSION

The main findings of this study were: (1) the large variability of plasma iron concentration in healthy neonatal foals, (2) the lack of statistically significant differences in plasma iron concentration in sick and SIRS affected foals relative to those in healthy foals, and (3) a negative correlation between plasma iron concentration and age for the first 2 weeks of life.

Neonatal foals in the stud control group (n = 135), only had a 24 hours range of age dispersion between all individuals, and their mean plasma iron concentration was 181.84 μg/dL, which is similar to the estimated average concentrations in neonatal foals of 1 day of age^14^; but presented a wider distribution (58.8‐305 μg/dL) compared to previously published plasma iron concentration in adults (105‐277 μg/dL).^2^, ^12^ In healthy foals at birth, plasma iron and ferritin concentrations are lower than in adult horses, followed by a rapid increase in the first 24 hours as a result of absorption of colostrum iron.^14^ Colostrum and milk are the major source of iron for neonatal foals with an average iron concentration of 0.79 mg/L in colostrum and 0.34 mg/L in milk.^17^ During the first 3 weeks of life, concentrations fall below concentrations of adults, reaching a nadir at 3 weeks of age.^18^ The same rapid changes occur after delivery in human newborns, when hemoglobin breakdown leads to storage of the iron by‐products for future erythrocyte production. Within the first week after delivery, hemoglobin and hematocrit values are higher and begin to drop in response to the higher ambient oxygen concentration ex utero.^13^ Based on previous studies of iron metabolism in foals^16^ where plasma iron concentration and several hematologic values changed rapidly during the first days of life in healthy foals we expected fluctuations depending on foals’ age; however, it was unexpected to observe a large variability of plasma iron concentrations in neonatal foals of a given age (ie, 24‐48 h). In addition, significant differences between healthy and SIRS foals were expected in this study but were not observed. Perhaps, grouping foals according to age (ie, 1‐3 days, 3‐7 days, and <14 days) would have helped us to better assess plasma iron dynamics.

In the studied population of neonatal foals, there was no significant difference in mean plasma iron concentrations between healthy foals and sick foals (*P* = .99). In equine medicine, previous reports suggest that plasma iron concentrations in adult horses are an acute and sensitive indicator of systemic inflammation.^2^, ^12^ In adult horses plasma iron concentration is considered decreased when values fall below the reference interval (105‐277 μg/dL).^2^ We expected to find lower iron plasma concentrations in sick foals compared to healthy foals, as occurs in the equine adult population because of the iron withholding mechanism. In the setting of infectious, inflammatory, and neoplastic diseases, a primitive defense mechanism of the organism is to withhold iron from microorganisms.^20^ The iron withholding defense system includes constitutive iron‐binding components such as transferrin, lactoferrin, and ovotransferrin, as well as the suppression of iron efflux from macrophages, reduction in plasmatic iron, and increased synthesis of ferritin by macrophages to accommodate iron from phagocytised lactoferrin iron.^21^ At baseline and in response to nutritional iron deficiency, infection, bleeding and pregnancy, hepcidin regulates iron metabolism.^22^ Hepcidin is potently induced by inflammation, predominantly by the cytokine IL‐6, and has shown to be essential for innate immunity to gram‐negative bacteria.^22^ This hormone controls iron flows into plasma through inhibition of the only known mammalian cellular iron exporter ferroportin.^23^ Hepcidin is feedback‐regulated by iron status and strongly modulated by inflammation and erythropoietic demand.^23^

Studies of plasma iron concentrations in neonatal foals with SIRS are sparse or nonexistent. The SIRS criteria have been defined in veterinary medicine by different authors.^3^, ^7^, ^23^, ^24^ The standard variables reported to define SIRS have been: (1) temperature, (2) heart rate (HR), (3) respiratory rate (RR), and (4) white blood cell counts (WBC) or presence of immature neutrophils. Comparison between various studies of SIRS in neonatal foals could be hampered given the differences in criteria and cutoff values relative to the definition of criteria of SIRS in neonatal foals. Some authors suggest different cutoff values for HR, RR, WBC in neonatal foals as well as including blood glucose and lactate concentrations^3^, ^9^, ^10^, ^25^ and others, include a fifth criteria; evidence of sepsis, cerebral hypoxia, ischemia, or trauma.^8^ The true clinical utility of the SIRS criteria in neonatal foals remains to be determined.^3^ In the present study a decision was made to use the original SIRS criteria rather than the neonatal SIRS criteria including glucose and lactate concentrations, because a higher sensitivity (60% versus 42%) to predict sepsis has been demonstrated.^3^ Although one of the weak points of the original SIRS criteria is that foals presenting only tachycardia and tachypnea could be misclassified in the SIRS group, in our population considering the updated neonatal SIRS criteria aforementioned, only 3 of 24 SIRS foals would be in this situation. Plasma iron concentrations for these neonatal foals were 345, 369.6, and 359.9 μg/dL, respectively. Using both SIRS criteria (original and updated) 14 of 47 sick neonatal foals would be classified in the SIRS group.

Although SIRS criteria are much simpler and faster screening tool for sepsis detection than sepsis scores, the clinician must be cognizant of the fact that SIRS can be caused by a number of causes other than infection such as trauma, burns, pancreatitis, ischemia, hemorrhage, and anaphylaxis.^5^, ^7^ In the present population of foals classified with the original SIRS criteria, no differences were seen in plasma iron concentration between SIRS and non‐SIRS sick foals. The expected finding would have been higher plasma iron concentrations in hospital controls and significantly lower plasma iron concentrations in neonatal foals with SIRS. Data on the incidence of SIRS and the usefulness of biomarkers of SIRS in veterinary medicine are scarce.^27^ Other markers of systemic inflammation currently validated in equine neonatology are plasma fibrinogen, serum amyloid A^27^, ^28^ and C‐reactive protein concentrations.^30^

In the study herein reported, surprisingly, different plasma iron concentrations resulted from each healthy control groups (hospitalized controls: 126.1 [99.2‐153.1] μg/dL and stud controls: 181.8 [171.3‐192.4] μg/dL, mean and [95% CI of the mean], respectively). It could be reasonable to think that sick dams can be hypogalactic,^17^ and as explained before, colostrum and milk are the major source of iron for newborns. Perhaps measuring plasma and colostrum/milk iron concentration of the mares and the amount of colostrum ingested would have been useful in explaining the differences between the control populations. On the other hand, these results could also be explained by the effect of the age (*P* < .001). Age differences between the stud control and healthy hospitalized foals (1.5 versus 4.5 days, respectively) could at least partly explain the differences observed in plasma iron concentrations (Figure 2). To rule out the age effect in future studies, we suggest classifying foals according to age (ie, 1‐3 days, 3‐7 days, and <14 days), in order to differentiate plasma iron concentrations in SIRS and non‐SIRS neonates as some other authors have done measuring age‐dependent plasma biochemical variables in neonatal foals.^31^

Another of the limitations of this study could be the absence of information regarding iron metabolism in the animals included, because, except for plasma iron concentration, no other variables related to iron metabolism were analyzed. Iron metabolism in equine medicine can be directly evaluated by measuring the amount of iron in the blood, including: plasma iron concentration, the capacity of the blood to transport iron by measuring transferrin or total iron binding capacity (TIBC) and the amount of iron storage measuring ferritin.^18^ Another limitation of this study could be the lack of a full hematology data from the stud control group. All samples were taken from healthy foals based on: normal foaling, normal physical exam, and serum immunoglobulin G concentration above 800 mg/dL within 24‐48 hours postpartum. Despite this limitation, we considered that a large homogeneous healthy group, with a daily close veterinary follow‐up was still of a great value for the study.

## 5 CONCLUSIONS AND CLINICAL RELEVANCE

In summary, plasma iron concentration has a negative moderate correlation with age in neonatal foals. Unlike what is reported in adult horses’ plasma iron concentration is not a useful early inflammatory marker to predict SIRS in sick neonatal foals. Based on results of this study plasma iron concentration in healthy neonatal foals has a larger variability compared to adults. Finally, plasma iron concentration is not a useful prognostic marker in this population.

## CONFLICT OF INTEREST DECLARATION

Authors declare no conflict of interest.

## OFF‐LABEL ANTIMICROBIAL DECLARATION

Authors declare no off‐label use of antimicrobials.

## INSTITUTIONAL ANIMAL CARE AND USE COMMITTEE (IACUC) OR OTHER APPROVAL DECLARATION

Authors declare no IACUC or other approval was needed.

## HUMAN ETHICS APPROVAL DECLARATION

Authors declare human ethics approval was not needed for this study.
